# Eupalinolide A inhibits cancer progression and induces ferroptosis and apoptosis by targeting the AMPK/mTOR/SCD1 signalling in non-small cell lung cancer

**DOI:** 10.3389/fphar.2025.1649314

**Published:** 2025-11-12

**Authors:** Yonghui Zhang, Chen Wang, Xiyang Zhang, Dilong Chen, Zhenni Ren, Haoning Yang, Nianhao Wang, Zumi Tang, Xiling Long, Lilian Zhao, Na Zhou, Junqi Zhang, Lei Li, Changxiao Hu, Feng Wang, Jiafeng Tang, Fulai Kang, Lianhong Pan, Yong Wei

**Affiliations:** 1 Chongqing Key Laboratory of Development and Utilization of Genuine Medicinal Materials in Three Gorges Reservoir Area, Chongqing Three Gorges Medical College, Chongqing, China; 2 Chongqing Three Gorges Medical College’s Medical-Engineering Integration Platform for Modernization of Traditional Chinese Medicine, Chongqing, China; 3 Chongqing Municipal Medical Products Administration, Chongqing, China; 4 Internal Medicine, Qianjiang District Traditional Chinese Medicine Hospital, Chongqing, China; 5 Key Laboratory of Intelligent Information Processing and Control, College of Electronic and Information Engineering, Chongqing Three Gorges University, Chongqing, China

**Keywords:** non-small cell lung cancer (NSCLC), Eupalinolide A (EA), AMPK-mTORsignalling pathway, ferroptosis, stearoyl-CoA desaturase 1 (SCD1)

## Abstract

**Background:**

Non-small cell lung cancer (NSCLC) is a common malignancy with limited efficacy of established therapies and a poor prognosis that often entails a high financial burden. Eupatorium lindleyanum DC. and its main component Eupalinolide A (EA) are clinically used for the management of chronic tracheitis. Therefore, the aim of this study was to investigate the effects of EA on NSCLC and its underlying molecular mechanisms.

**Methods:**

The effect of EA was assessed on A549 and H1299 tumour cells by flow cytometry, qRT-PCR, western blotting, TUNEL assay, molecular docking, RNA sequencing, non-target metabolomics experimental methodology, and phosphoproteomics to explore the molecular pathways and specific targets. A mouse tumour xenograft model was used to evaluate its effect *in vivo*.

**Results:**

EA inhibited cell proliferation by arresting the cell cycle at the G2/M phase (increasing the proportion of G2-phase cells in A549 cells from 2.91% to 21.99% and their proportion in H1299 cells from 8.22% to 18.91%). It also promoted apoptosis (increasing the total apoptotic rate in A549 cells from 1.79% to 47.29% and that in H1299 cells from 4.66% to 44.43%) as well as ferroptosis (increasing ROS production by 2.46-fold in A549 cells and by 1.32-fold in H1299 cells). This mechanism was mediated by the modulation of the synthesis of unsaturated fatty acids through the downregulation of stearoyl-CoA desaturase 1 (SCD1) expression, which was reduced by 34% in A549 cells and by 48% in H1299 cells. Notably, the supplementation of oleic acid counteracted the inhibitory effect of EA on cell proliferation and migration. These effects of EA were due to the involvement of the AMPK- mTOR signalling pathway. EA treatment (25 mg/kg) markedly inhibited tumour growth *in vivo* in a xenograft model; both tumour weight and tumour volume decreased by more than 60% without significantly affecting the body weight of tumour-bearing mice.

**Conclusion:**

EA inhibited the proliferation and migration of NSCLC cells by the modulation of lipid metabolism through the activation of the ROS-AMPK-mTOR-SCD1 signalling pathway. Therefore, EA might be considered as a promising candidate in the development of therapeutics against NSCLC.

## Introduction

Non-small cell lung cancer (NSCLC) is a highly aggressive malignancy worldwide with a high mortality rate (Hendriks et al., 2024). The current treatment strategies for lung cancer include surgical resection, radiotherapy, targeted therapy and chemotherapy but despite these multiple treatment options, patient’s prognosis is not ideal (Li et al., 2023a; The Lancet, 2024). Chemotherapy is one of the most common treatments, although traditional chemotherapeutic drugs have common limitations, such as poor targeting, low bioavailability and tumour recurrence caused by drug resistance, which greatly affect their effectiveness (Zhu et al., 2021). Therefore, it is of the utmost importance to find new drugs to improve the therapeutic outcomes of NSCLC.

Metabolic reprogramming is one of the landmarks in the development and progression of tumours (Feng et al., 2024; Nam et al., 2024). Lipid metabolism regulates multiple biological processes in tumour cells, including the mediation of signalling transduction, the biosynthesis of cells, and the promotion of cell growth (Pascual et al., 2024; Wang et al., 2024). The *de novo* fatty acid synthesis is an emerging key factor in cancer malignancy that ensures the energetic and biosynthetic requirements of rapidly proliferating tumour cells (Tu et al., 2025). Therapies targeting lipid metabolism-related elements inhibit tumour cell proliferation and migration and tumour immune escape (Sena and Denmeade, 2021), thus providing a potential new therapeutic method.

The continuous exploration and development of traditional Chinese medicine has led to its application with herbs that effectively reduce the toxicity of radiotherapy and chemotherapy, improve the clinical prognosis of patients, and attract more and more attention in cancer treatment (Pan et al., 2022; Zhang et al., 2022a). The active extracts of Chinese herbal plants are effective in the treatment of tumours, including NSCLC. For example, erianin exerts an anti-cancer effect by inducing ferroptosis, which further inhibits lung cancer cell biological behaviour (Chen et al., 2020). Evodiamine suppresses NSCLC cell growth and induces apoptosis by regulating the MUC1-C/programmed death 1 (PD-L1) axis (Jiang et al., 2020). Nitidine chloride exerts its anti-tumour effect by inducing caspase 3/GSDME-dependent pyroptosis in lung cancer (Yu et al., 2022). Eupalinolide A (EA) is a sesquiterpene lactone constituent isolated from the traditional Chinese medicine *Eupatorium lindleyanum* DC. (Wang X. et al., 2020) with several pharmacological activities, including anti-inflammatory, antimicrobial, and anti-tumour effects (Jiang et al., 2024; Zhang et al., 2022b; Li et al., 2023b). However, despite its several pharmacological activities, the effect and in-depth mechanism of EA in lung cancer are lacking, with no literature reports.

Therefore, this study aimed to determine the effect of EA against NSCLC and its underlying molecular mechanisms as well as investigate the potential ability of EA to regulate the lipid metabolism of NSCLC cells. Our results demonstrated that EA exerted anti-NSCLC activity *in vitro* and *in vivo*. It regulated the biosynthesis of unsaturated fatty acids by reducing oleic acid expression in lung cancer cells, consequently modulating apoptosis and ferroptosis by the activation of the ROS-AMPK-mTOR- stearoyl-CoA desaturase 1 (SCD1) signalling pathway. In addition, it inhibited the proliferation and migration while promoting the apoptosis of lung cancer cells. Thus, this is the first study demonstrating that EA might be a potential drug candidate in the treatment of NSCLC, since it worked by targeting apoptosis and ferroptosis through the regulation of lipid metabolism. Hence, this work might contribute to the identification of new therapeutic targets for NSCLC therapy, potentially overcoming the resistance to traditional chemotherapeutic drugs in patients with NSCLC.

## Materials and methods

### Cell culture and treatment

The lung adenocarcinoma epithelial cell line A549 and the human non-small cell lung carcinoma cell line H1299 were obtained from Shanghai Zhong Qiao Xin Zhou Biotechnology Co., Ltd., RPMI-1640 (Gibco, NY, NY, USA) was used to culture A549 cells and Dulbecco’s modified Eagle’s medium (DMEM) (Gibco, NY, NY, USA) to culture H1299 cells, both supplemented with 10% fetal bovine serum (FBS) (Cellmax, Beijing) and 1% penicillin/streptomycin (Invitrogen, CA, USA). EA was obtained from Must (A0659, Sichuan, China). A549 and H1299 cells were treated with EA according to the protocols described in each paragraph.

### Cell morphology

A549 and H1299 cells were cultured in 6-well plates and treated with EA (10, 20, and 30 μM) for 24 h. Images were taken using a phase-contrast microscope (Olympus, Tokyo, Japan).

### Cell viability assay

The viability of A549 and H1299 treated with EA was assessed using Cell Counting Kit-8 (CCK8) assay (Suzhou, China). A549 and H1299 cell lines were seeded and incubated until reaching a confluence of approximately 90%. Next, cells were seeded into 96-well plates at a concentration of 5 × 10^3^ cell/well and treated with EA (10, 20, or 30 μM) for 48 h. CCK8 (10 μL) was added into each well, and the cells were incubated at 37 °C for 1.5 h. The absorbance was measured at 450 nm and recorded.

### EdU assay

Cells were seeded into a 6-well plate and treated with EA. Then EdU (10 μM) wasadded into each well and the cells were incubated for 2 h to measure their proliferation. Cells were fixed with 4% paraformaldehyde for 0.5 h and 0.1% Triton X-100 for 0.5 h to enhance their accessibility for further analysis. Cells were washed, endogenous peroxidase blocking solution was added and the cells were incubated for 0.5 h. Next Click reaction solution was added and the cells were incubated for 0.5 h. Streptavidin-HRP working solution and 3,3′-diaminobenzidine tetrahydrochloride (DAB) chromogenic solution were added into each well and images were taken under an ordinary optical microscope. EdU detection was carried out using the BeyoClick™ EdU Cell Proliferation Kit containing DAB (C0085S, Beyotime, China).

### Tumour xenografts

Animal experiments were approved by the Ethics Committees of Chongqing Three Gorges Medical College (SXYZ-A-2501-0001) and performed accordingly. Four-week-old nude mice were purchased from the laboratory animal research centre of Chongqing Medical University and subjected to acclimatization for 1 week in an SPF environment. They were randomly divided into three groups: A549 cells were treated with dimethylsulfoxide (DMSO) or EA (25 and 50 mg/kg), cell concentration was adjusted to 1 × 10^6^ in 100 μL matrigel (Corning, USA) and they were injected into both armpits of the mice. Tumours became visible after 1 week. Theexperiment index of nude mice was measured and recorded every 3 days, including weight and tumour volume. Tumour volume was calculated as follows: volume = tumour length × width^2^ × π/6 (Pan et al., 2021). Mice were euthanized and tumours were collected for further analysis.

### Cell migration analysis

Cells were cultured in 6-well plates until reaching 70% confluence and a scratch was made using a sterile pipette tip along a horizontal line across the centre of the well to generate a cell-free gap. Cells were gently washed using phosphate-buffered saline (PBS) to remove cell debris and medium containing appropriate supplements was added. Then EA (0, 10, 20, or 30 μM) was added and the cells were incubated for 24 h. Images were taken at time zero (T0) and after 6, 24 and 48 h to assess wound closure.

Migration was also assessed by transwell assay. A transwell 24-well plate (8 μm, Corning, USA) was used to create two chambers, 1 × 10^4^ cells were dissolved in medium containing 1% serum and seeded into the upper chamber, while medium containing 10% FBS was placed in the lower chamber and used as a chemoattractant compound. Then, EA (0, 10, 20, or 30 μM) was added to the cells, which were incubated for 24 h. Cells in the upper chamber were washed 3 times with PBS, wiped using a cotton swab, fixed with 4% paraformaldehyde, stained with 0.5% crystal violet, washed with PBS and dried. Images were taken under a microscope (Olympus, Japan).

### Western blot

Cells were lysed using RIPA buffer (Beyotime, China). The lysate was heated at 100 °C for 5 min. Proteins were separated by 10% SDS-PAGE gel, ensuring high resolution and accuracy. Then, proteins were transferred onto a polyvinylidene difluoride membrane. The membrane was incubated in 5% milk for 2 h to block non-specific binding sites. The membrane was incubated with the following primary antibodies at 4 °C for 12 h: CDC25C (Proteintech; 1:1,000; cat. no. 66912-1), cyclin B1 (Proteintech; 1:1,000; cat. no. 67686-1), BAX (Proteintech; 1:1,000; cat. no. 60267), SCD-1 (Proteintech; 1:1,000; cat. no. 28678), GPX4 (Abcam; 1:1,000; cat. no. ab125066), HO-1 (Abcam; 1:1,000; cat. no. ab13248), SLC7A11 (CST; 1:1,000; cat. no. 12691S), PTGS2/COX2 (Abways; 1:1,000; cat. no. CY8852), BCL2 (Abways; 1:1,000; cat. no. CY5032), p-AMPK (Abclonal; 1:1,000; cat. no. APO871), AMPK (Abclonal; 1:1,000; cat. no. A1229), p-mTOR (CST; 1:1,000; cat. no. 5536T), mTOR (Immunoway; 1:1,000; cat. no. YT2913), and β-actin (Zhongshan Goldenbridge Bio; 1:1,000; cat. no. TA-09). Next, the membrane was incubated with HRP-conjugated secondary antibodies (A0216 and A0208, Beyotime; 1:1,000) for 2 h at 37 acy. inally, proteins were visualized using an ECL system (Biorad, USA). The expression of CDC25C, cyclin B1, BAX, SCD-1, GPX4, HO-1, SLC7A11 and BCL2 was normalized to t. no. T.

### Transcriptome sequencing experiment

Cells were treated with DMSO or EA (20 μM), subsequently harvested in RNA extraction Reagent (Tiangen, Beijing) and stored at −80 °C, then delivered to Shanghai Bioroot Biological Technology Co., Ltd., for transcriptome sequencing analysis using the Illumina sequencing platform. Raw sequencing data were subjected to a rigorous filtering, and filtered sequences were mapped to the human reference genome. The expression of individual genes was quantified based on the alignment results, enabling the performance of subsequent differential expression analysis, functional enrichment analysis, and clustering analysis on the samples. This comprehensive workflow provided a robust framework for investigating transcriptional changes in NSCLC cell lines treated with EA or DMSO.

### Non-target metabolomics experimental methodology

Cells were seeded into a 6-well plate and treated with DMSO or EA (20 μM) for 24 h. Next, the culture medium was removed, and the cells were washed twice with an appropriate amount of pre-cooled PBS. Cells were collected and stored in liquid nitrogen or at −80 °C refrigerator and subsequently delivered to Shanghai Bioroot Biological Technology Co., Ltd., for metabolic analysis. Metabolite extraction and LC-MS/MS detection were performed, along with ProteoWizard and R Programming Language based on Various forms (X) of chromatography mass spectrometry (XCMS), for peak detection, extraction, alignment, and integration. Ten samples were collected from each group. Figure 6A shows the metabolomics experimental method flow chart. Cells were collected from cell culture flasks according to the requirements of metabolomics for collecting samples. LC-MS/MS Orbitrap mass spectrometer or tandem Orbitrap mass spectrometer was used to detect metabolites in samples by retention with metabolites in the local database. Metabolomics usually used strict OPLS-DA VIP >1 and *P* < 0.05 as the screening criteria for significant differential metabolites.

### Flow cytometry analysis

Cell cycle assay was performed on cells treated with DMSO or EA for 24 h and subsequently harvested for flow cytometry analysis. Cells were washed 3 times with sterile PBS, and treated with 75% ethanol at 4 °C overnight to ensure proper preservation. Post-fixation was performed by washing the cells 3 times with pre-cooled PBS to remove residual ethanol. Subsequently, the cells were incubated with propidium iodide (PI) and RNaseA (PI: RNaseA = 9:1) in a 500 μL mixture of PBS for 1 h to perform the DNA staining and RNA digestion. Finally, cells were analysed using the DxFLEX flow cytometer (Beckman, Germany).

Cell apoptosis assay was performed on cells treated with DMSO or EA for 48 h, collected and rinsed twice with sterile PBS. Subsequently, cells were resuspended in binding buffer and Annexin V-FITC (FAK015, NeoBioscience, Beijing) was added to bind with the cell membrane for 0.5 h. Next, PI was added to bind the nuclear DNA, and the cells were analysed using the DxFLEX flow cytometer (Beckman, Germany).

ROS were detected in cells treated with DMSO or EA for 48 h, collected and resuspended in diluted DCFH-DA solution. Then, cells were incubated at 37 ding buffer and Annexin V-FITC (FAK015, NeoBioscience, Beijing) and analysed using the DxFLEX flow cytometer (Beckman, Germany).

### Tunel assay

A549 and H1299 cells were seeded into 6-well plate and treated with DMSO or EA 10 μM or 20 μM for 48 h. Cells were fixed with 4% paraformaldehyde and permeabilized with 0.1% Triton X-100. TUNEL reagent (Dalian Bergolin Biotechnology Co., Ltd.) was added and the cells were incubated at 37 °C for 2 h. DAPI (100 μL) wasadded into each well and the cells were incubated for 20 min. Slides were analysed under a microscope (Olympus, Japan).

### Transmission electron microscopy (TEM)

Cells were treated with DMSO or EAand fixed with 2.5% glutaraldehyde solution at 4 °C overnight. The fixative was removed, cells were washed 3 times with pre-cooled PBS and 1% osmic acid solution was added for further fixation. Samples were dehydrated using ethanol solutions (30%, 50%, 70%, 80%, 90% and 100%), 20 min per solution, then dehydrated with pure acetone for 0.5 h. Samples were treated with different proportions of liquids (acetone:resin = 3:1, acetone:resin = 1:1, acetone:resin = 1:1), 3 h per solution. Finally, they were treated with 100% resin for 12 h. Samples were placed in a mold and subjected to gradient heating (35 °C, 60 °C and 80 °C an 5 h each step, resulting in well-embedded samples. After coarse trimming, samples were cut into 80 nm-thick sections using an ultrathin microtome (Leica, Germany). Sections were stained with acetic acid dioxy dye solution (15 min) and lead citrate (5 min), dried, and then ready for microscopic observation.

### Fe^2+^ fluorescence assay

Cells were treated with DMSO or EA for 48 h, washed 3 times with serum-free medium, 1 μM FerroOrange (Dojindo, Japan) working solution was added into each well and cells were incubated at 37 °C for 0.5 h. Finally, samples were observed under a fluorescence microscope (Olympus, Japan).

### Gene expression analysis

Cells were treated with DMSO or EA for 48 h, and total RNA was extracted using the TRnaZol RNA Kit (New Cell and Molecular Biotech Co., Ltd., Suzhou). Five mg of this isolated total RNA was used as a template to perform cDNA synthesis using the All-In-One 5X RT MasterMix (Applied Biological Materials Inc., Suzhou, China), following the manufacturer’s protocol. Subsequently, qRT-PCR was performed on the ABI 7500 Real-Time PCR Instrument (ABI, USA), using the BlasTaqTM 2X qPCR MasterMix (Applied Biological Materials Inc., Suzhou) to ensure the accurate and sensitive detection of gene expression. The expression of target mRNAs was normalized against β-actin mRNA expression in the same sample, providing a reliable internal control for quantitative analysis. The primers used are listed in Table 1.

**TABLE 1:** Primer sequences for real-time qRT-PCR.

Gene name	Forward primer	Reverse primer
*GPX4*	GAG​GCA​AGA​CCG​AAG​TAA​ACT​AC	CCG​AAC​TGG​TTA​CAC​GGG​AA
*HMOX1*	CAG​TGT​TTG​GAC​GGA​ACA​GAT	GGT​TGG​CAA​GAA​GGA​GCT​AAC
*SLC7A11*	TTG​CTG​GGC​TGA​TTT​ATC​TTC	GGG​TCC​GAA​TAG​AGG​GAA​AG
*PTGS2*	CTG​GCG​CTC​AGC​CAT​ACA​G	CGC​ACT​TAT​ACT​GGT​CAA​ATC​CC
*CDC25C*	TCT​TGC​TCA​GAG​GCC​GTA​AC	GCA​ACG​TTT​TGG​GGT​TCC​TC
*Cyclin B1*	GCA​CTT​CCT​TCG​GAG​AGC​AT	TGT​TCT​TGA​CAG​TCC​ATT​CAC​CA
*SCD1*	TCC​GAC​CTA​AGA​GCC​GAG​AA	TGG​GCA​GCA​CTA​TTC​ACC​AG
*β-actin*	GCCTCGCCTTTGCCGAT	AGG​TAG​TCA​GTC​AGG​TCC​CG

### Molecular docking

Molecular Operating Environment software was used to dock EA (target molecule) with SCD1 protein and the binding free energy was calculated. The docking results were further analysed using the Discovery Studio 4.5 Client software to study the intermolecular interaction abilities.

### Plasmid construction and transfection

Cells were transfected with the expression vector (ov-SCD1) using translipofectamine (Beyotime, Shanghai). Briefly, cells were cultured and a total of 125 µL DMEM with 2.5 µg plasmid DNA was added into each sample, then added lipo8000 (4 μL; Beyotime, Shanghai) was added to each sample, and cultivate for 24 in the incubator. Cells were collected and qRT-PCR was performed to detect SCD1.

### Phosphorylation array

Cells were treated with DMSO or EA for 24 h, and the level of phospho kinases was determined using the Phosphorylation Array C55 kit (RayBiothech, China) according to the manufacturer’s protocol.

### Histological analysis

Tumours were harvested and fixed in 4% paraformaldehyde for 2 days to preserve the tissue architecture. Then, the tumour was embedded in paraffin and cut into consecutive 8 mm-thick slides using a microtome. The slides were stained with haematoxylin and eosin (H&E), and cell morphology and tissue structure were observed under the microscope (Olympus, Japan).

### Statistical analysis

Statistical analysis was performed using Graphpad 8.0. Two groups were compared using Student’s t-test. Results were presented as mean ± S. D of at least three independent experiments. *P* < 0.05 was considered statistically significant.

## Results

### EA inhibits A549 and H1299 cell viability *in vitro*

**FIGURE 1:**
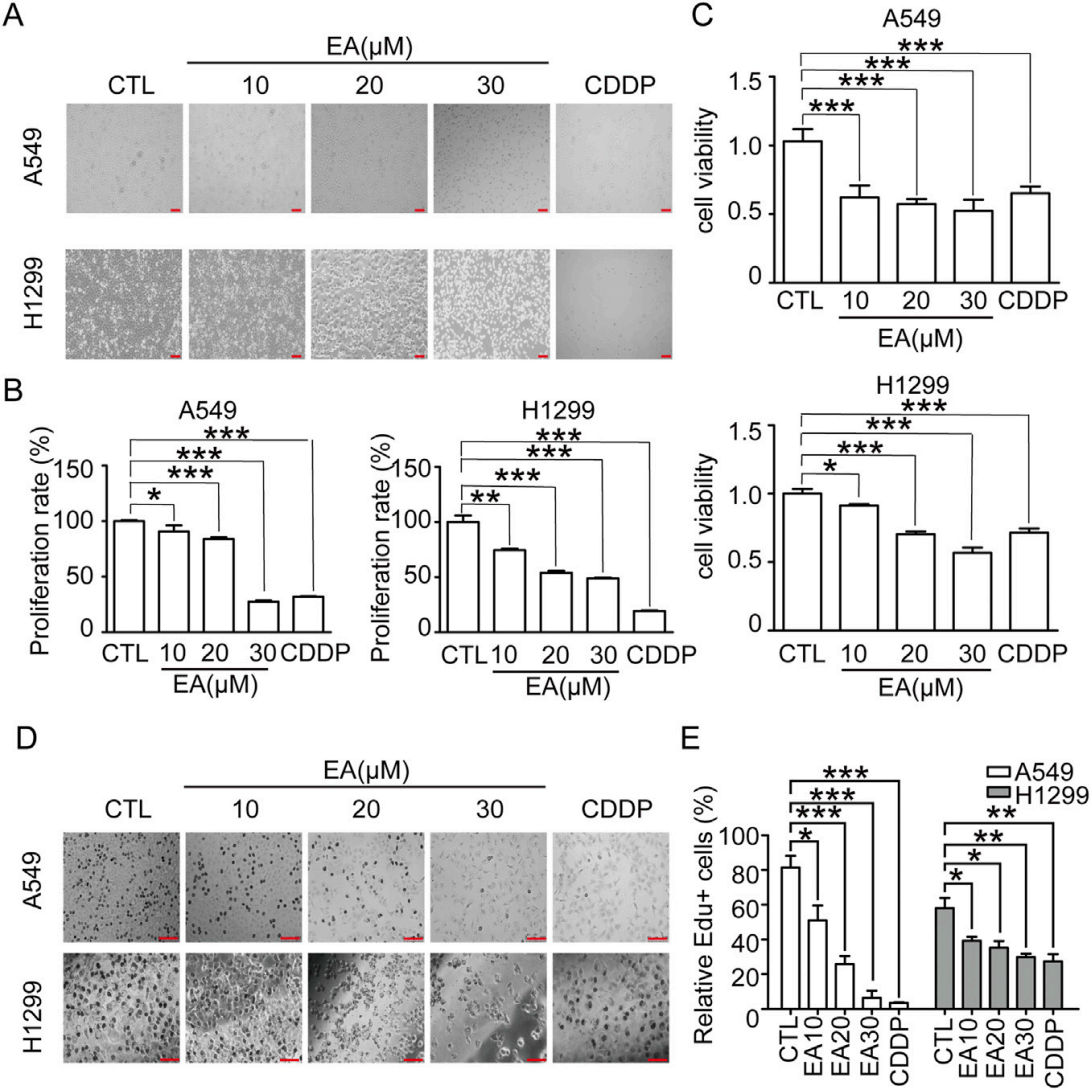
EA suppresses tumor growth in the human non-small cell lung cancer cells. **(A)** The morphology of A549 and H1299 cells, scale bar = 100 μm. **(B)** Relative numbers of A549 and H1299 cells. Cell numbers of DMSO-treated group were regarded as 100% n = 5. **(C)** Viability of A549 and H1299 cells n = 6. **(D,E)** EdU staining of A549 and H1299 cells after EA and CDDP treatment for 24 h and quantitation of the percentage of EdU-positive cells. Scar bar = 100 μm n = 5. *p < 0.05, **p < 0.01, and ***p < 0.001 compared with the Control group.

Cell treatment with various concentrations of EA and 30 μM cisplatin for 24 h led to a remarkable morphological alteration and a dose-dependent reduction in cell number compared to the cells treated with DMSO as control (Figures 1A,B). EA treatment and cisplatin significantly reduced cell proliferation, as shown in Figure 1C. This outcome underlined the potent suppressive effect of EA on NSCLC cell proliferation. EdU staining confirmed the above results, since a remarkable decrease in the proportion of EdU-positive cells was observed in the EA-treated group (Figures 1D,E), indicatinga substantial inhibition of NSCLC cell proliferation. Collectively, our comprehensive findings demonstrated that EA exerted a potent inhibitory effect on NSCLC proliferation *in vitro*.

### EA suppresses cell migration in A549 and H1299 cells by modulating epithelial-mesenchymal transition (EMT)

**FIGURE 2:**
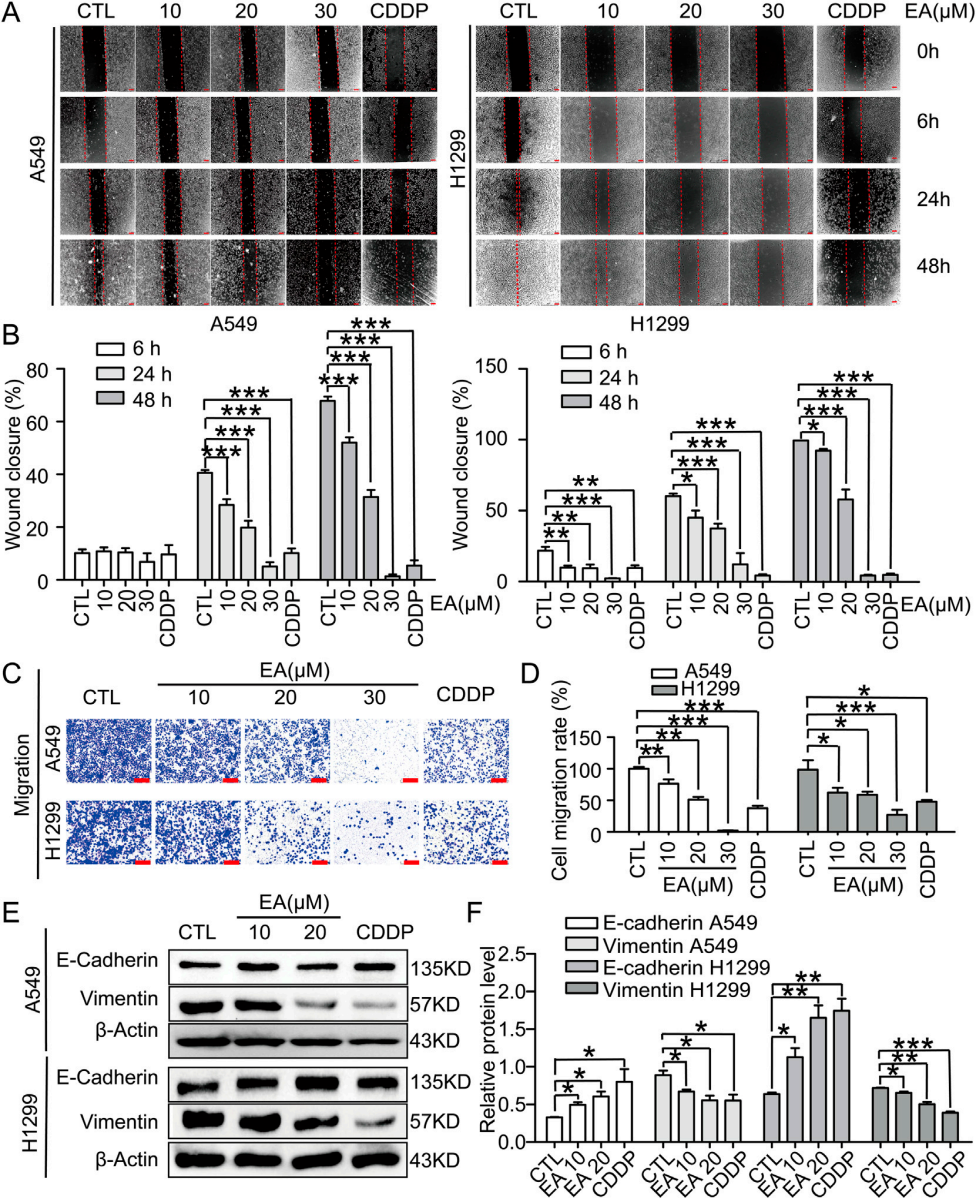
EA inhibits cell migration in human non-small cell lung cancer cells. **(A)** The migration by wound-healing assay of A549 and H1299 cells after treating with DMSO, 10, 20, 30 μM EA and CDDP for the indicated time, Scale bar = 100 μm. **(B)** The effect of EA on the wound closure in A549 and H1299 cells n = 5. **(C)** Transwell migration assays of A549 and H1299 cells treated with DMSO, 10, 20, 30 μM EA and CDDP for 24 h n = 5. **(D)** The statistical analysis has been presented in histograms, and the migration rates were normalised by the proliferation rate. **(E,F)** Western blot analysis of the E-Cadherin and Vimentin protein levels at 24 h in A549 and H1299 cells respectively n = 3. *p < 0.05, **p < 0.01, and ***p < 0.001 compared with the Con group.

In this study, scratch assay and transwell migration assay were performed to assess the effect of EA on A549 and H1299 cells, resulting in consistent outcomes in both assays. Our findings, demonstrated the inhibitory effect of EA on cell migration, as evidenced by the remarkable differences compared to the DMSO-treated controls (Figures 2A,B). The transwell migration assay further confirmed the above results, revealing a concentration-dependent reduction in the migration of cells after EA treatment (Figures 2C,D). This suppression was accompanied by an alteration in the expression of key EMT markers. Indeed, an upregulation of E-cadherin expression in an EA concentration and time-dependent manner was observed, whereas the expression of vimentin was inhibited (Figures 2E,F). These alterations collectively indicated that EA effectively reversed the EMT process in A549 and H1299 cells.

### EA regulates the differentially expressed genes and pathway enrichment in A549 and H1299 cells

**FIGURE 3:**
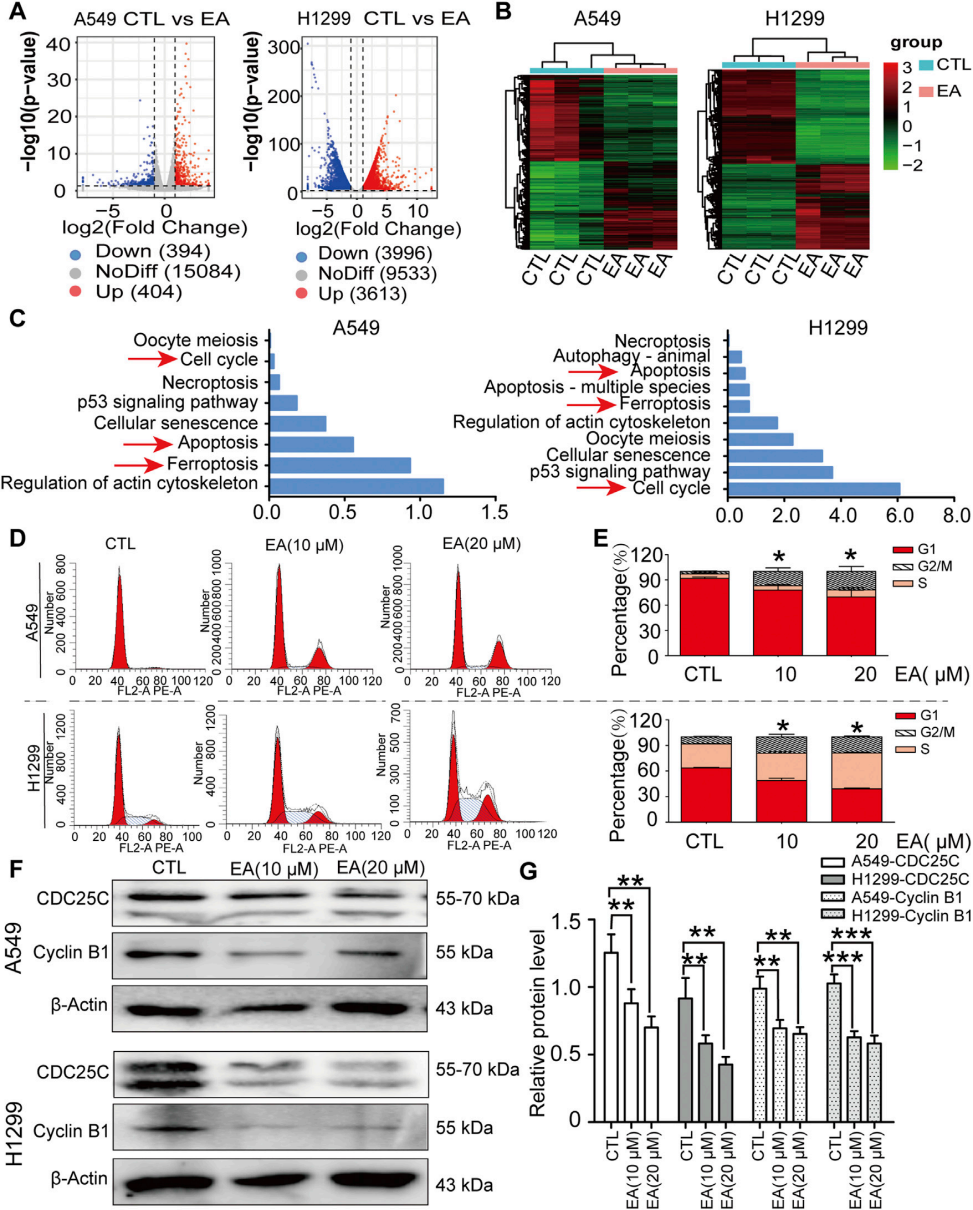
EA induces cell cycle arrest at G2/M phase in human non-small cell lung cancer cells. **(A)** Volcano plot of differentially expressed genes after EA treatment. **(B)** Cluster analysis of differentially expressed genes after EA treatment. **(C)** KEEG analysis of genes in A549 and H1299 cells. **(D)** Cell cycle of A549 and H1299 cells analyzed by flow cytometry. **(E)** Percentage of A549 and H1299 cells in different phases n = 3. **(F)** Representative Western blots of cell cycle-related proteins, CDC25C and Cyclin B1. **(G)** Densitometry of Western blots in panel n = 6. *p < 0.05, **p < 0.01, and ***p < 0.001 compared with the Control group.

Transcriptomics analysis was performed to explore the underlying mechanisms regulating the effect of EA. The results on A549 cells showed that 404 genes were upregulated and 394 were downregulated. In contrast, the H1299 cell line showed 3,613 upregulated genes and 3,996 downregulated genes after the treatment with EA (Figure 3A). Cluster analysis was performed to understand the classification of gene expression patterns, revealing the genes that increased or decreased in response to EA. Upregulated genes are shown in red, while downregulated genes are shown in green (Figure 3B).

The functional implications of these transcriptional shifts were investigated using KEGG enrichment analysis to discover the most relevant pathways (Figure 3C). Among the many pathways involved, our attention was focused on cell cycle, apoptosis, and ferroptosis, pathways, thus suggesting the potential of EA to regulate cell fate through these critical signalling pathways.

As regards cell cycle, our results showed an arrest in the G2/M phase after EA treatment (Figures 3D,E). Thus, the expression of CDC25C and cyclin B1, which are two proteins that regulate the cell cycle, was further assessed to analyse the mechanism regulating the arrest of the cell cycle. Our results confirmed that these proteins were indeed significantly decreased by EA, adding another evidence on the effect of EA against lung cancer (Figures 3F,G).

### EA induces apoptosis in A549 and H1299 cells

**FIGURE 4:**
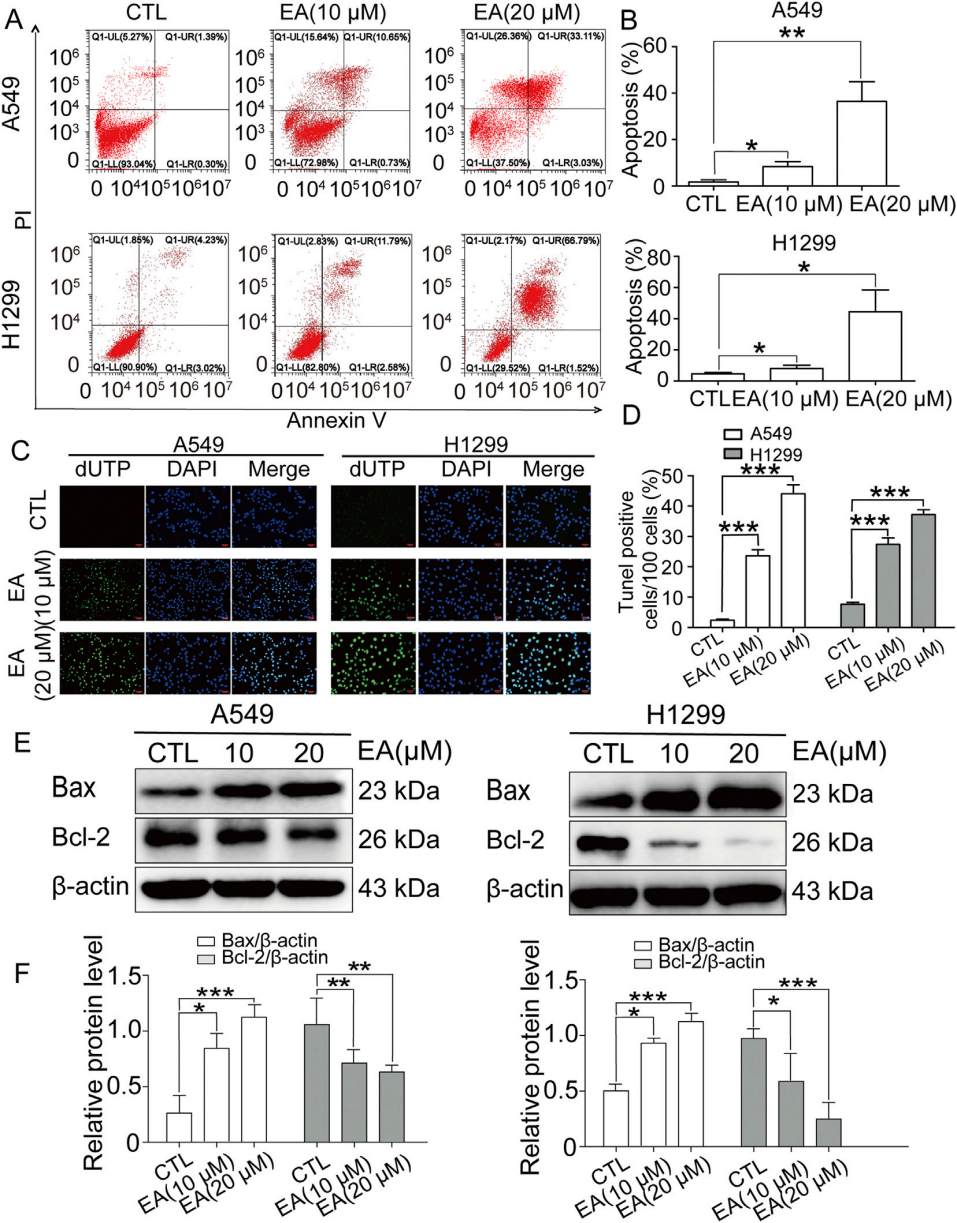
EA induces cell apoptosis in human non-small cell lung cancer cells. **(A)** Apoptosis analyzed by Annexin V/PI staining. **(B)** Apoptotic rates of A549 and H1299 cells n = 6. **(C)** The apoptosis-positive cells of A549 and H1299 cells after EA treatment for 24 h by TUNEL staining (×100). **(D)** Quantification of TUNEL-positive cells in **(C)** n = 5. **(E)** Representative Western blots of cell apoptosis proteins, Bax and Bcl-2. **(F)** Densitometry of Western blots in panel. *p < 0.05, **p < 0.01, and ***p < 0.001 compared with the Control group.

A clear induction of apoptosis was observed in both cells by flow cytometry (Figures 4A,B). Our findings were corroborated by TUNEL assay on A549 and H1299 cells under the same EA treatment. Indeed, TUNEL staining demonstrated a concentration-dependent increase of apoptotic cells under EA treatment (Figures 4C,D). Next, the expression of the key apoptotic regulators Bax and Bcl-2 was assessed to unravel the molecular pathways mediating EA-induced apoptosis. Our results revealed a significant downregulation of Bcl-2 and an upregulation of Bax in both A549 and H1299 cells after EA treatment, as compared to untreated controls (Figures 4E,F). These findings collectively suggested that EA exerted its apoptotic effects in NSCLC cells by regulating the pro and anti-apoptotic factors, thereby promoting programmed cell death.

### EA induces the ferroptosis of A549 and H1299 cells

**FIGURE 5:**
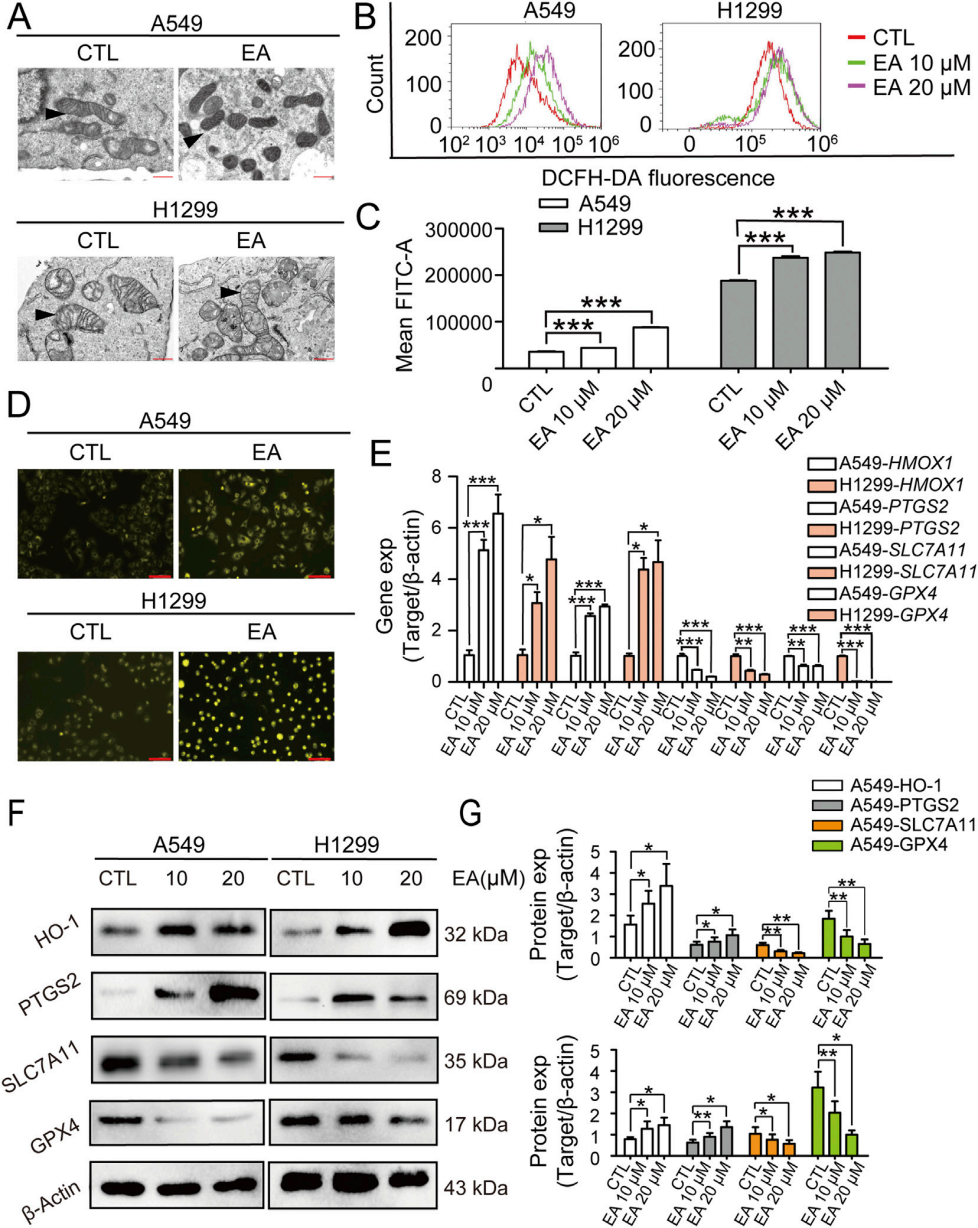
EA inhibits the proliferation of non-small cell lung cancer cells by regulating ferroptosis. **(A)** Electron microscopy observation of mitochondrial morphology in A549 and H1299 cells after EA treatment. **(B,C)** Flow cytometry detection of reactive oxygen species (ROS) in A549 and H1299 cells treated with different concentrations of EA n = 3. **(D)** Fe^2+^ expression in A549 and H1299 cells detected by immunofluorescence after EA administration. Scale bar = 100 μm. **(E)** The expression levels of ferroptosis-related genes in A549 and H1299 cells treated with different concentrations of EA, as detected by qRT-PCR n = 3. **(F)** Western blot was used to detect the changes of protein expression in A549 and H1299 cells treated with different concentrations of EA n = 6. **(G)** Densitometry of Western blots in panel. *p < 0.05, **p < 0.01, and ***p < 0.001 compared with the Control group.

The transcriptomefindings related to ferroptosisin A549 and H1299 cells under EA treatment were furtherconfirmed by the examination of their ultrastructure using TEM. TEM images revealed distinct morphological alterations in EA-treated cells, showing a reduced mitochondrial size associated with an increased membrane density, hallmarks indicative of ferroptosis (Figure 5A). These findings were corroborated by ROS generation in both A549 and H1299 cells following EA exposure using the DFCH-DA probe. Our results demonstrated a significant concentration-dependent increase in ROS levels after EA treatment (Figures 5B,C). Moreover, fluorescence microscopy analysis revealed an accumulation of Fe^2+^ ions in both A549 and H1299 cells, further supporting the induction of ferroptosis (Figure 5D). Subsequently, the expression of ferroptosis-associated genes revealed that EA treatment induced a significant upregulation of HMOX1 and PTGS2 genes, while it downregulated SLC7A11 and GPX4, key regulators that counteracted ferroptosis (Figure 5E). These results were confirmed at a protein level, showing the same results of protein expression of the above genes (Figures 5F,G), confirming the above discovery. Collectively, these results strongly suggested that EA induced ferroptosis in A549 and H1299 cells.

### EA modulates the lipid metabolism pathways in A549 cells

**FIGURE 6:**
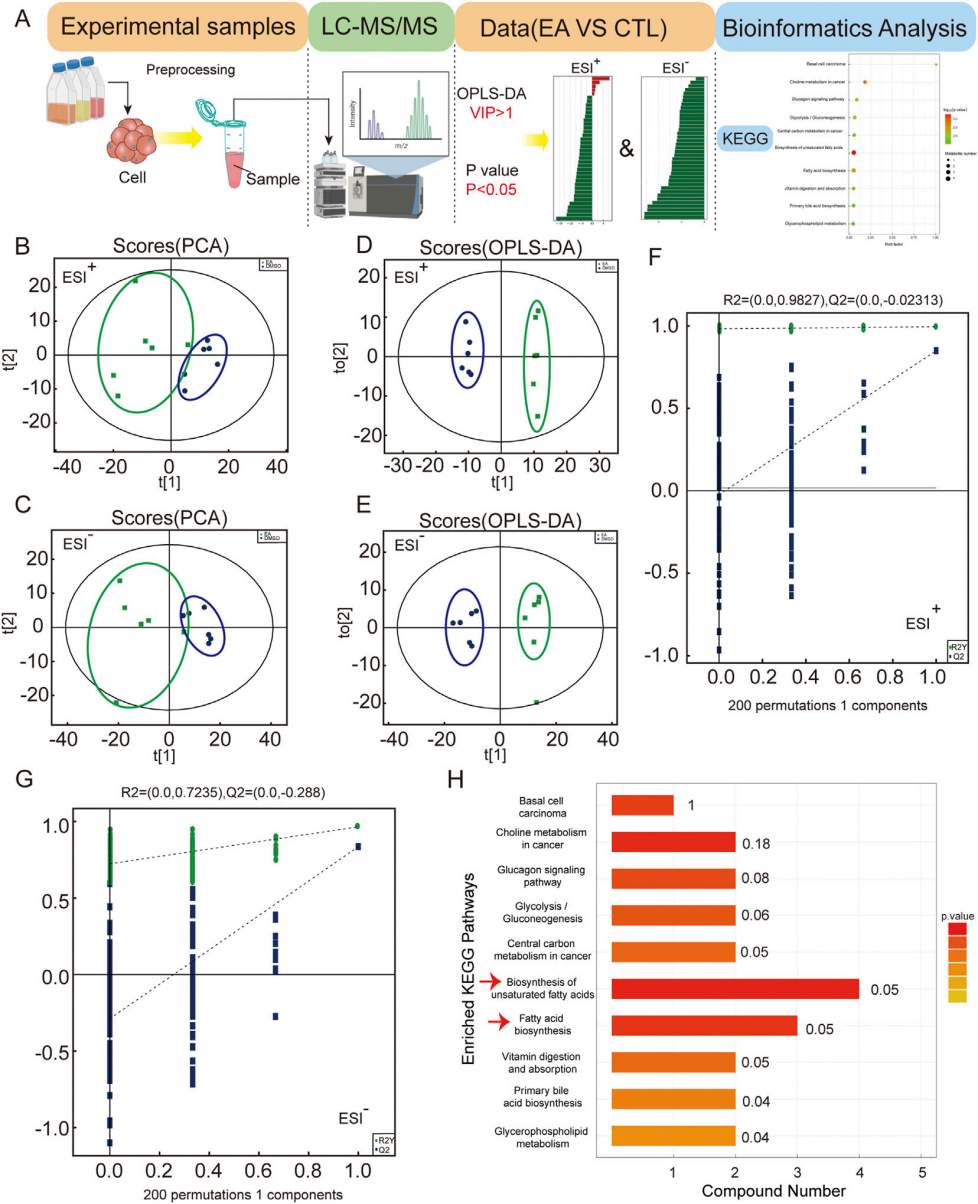
Metabolomic analysis reveals EA’s impact on non-small cell lung cancer metabolism via unsaturated fatty acid biosynthesis. **(A)** Untargeted metabolomics data analysis workflow. **(B,C)** Principal component analysis (PCA) of global samples in positive and negative ion modes. **(D,E)** Principal component analysis (OPLS-DA) of global samples in positive and negative ion modes. **(F,G)** Permutation test of OPLS-DA for positive and negative ion modes. **(H)** KEGG analysis enriched pathways.

The differential metabolites were subjected to enrichment analysis to obtain an enrichment pathway map. The data were subjected to PCA and OPLS-DA analysis. PCA method was used to detect the overall distribution trend and the difference between groups. PCA scores of positive and negative ion modes are shown in Figures 6B,C, and OPLS-DA score plot is shown in Figures 6D,E. The permutation test was further used to test the model to avoid the over-fitting and ensure the validity of the model. Figures 6F,G showed the permutation test diagram of the OPLS-DA model in the sample comparison group. R2 and Q2 values in the model were decreased with the decreasing of the permutation retention, indicating no overfitting phenomenon and the robustness of the model. Supplementary Figures 2A,B show the significant expression of different metabolites. The overall changes of KEGG metabolic pathways were analysed to rank the top ten signalling pathways, and the results showed that the biosynthesis of unsaturated fatty acids and fatty acid biosynthesis pathways were significantly altered, as shown in Figure 6H. Which metabolites were changed when the cells were treated with EA?

### EA regulates the cell biological behaviour by reducing monounsaturated fatty acids level

**FIGURE 7:**
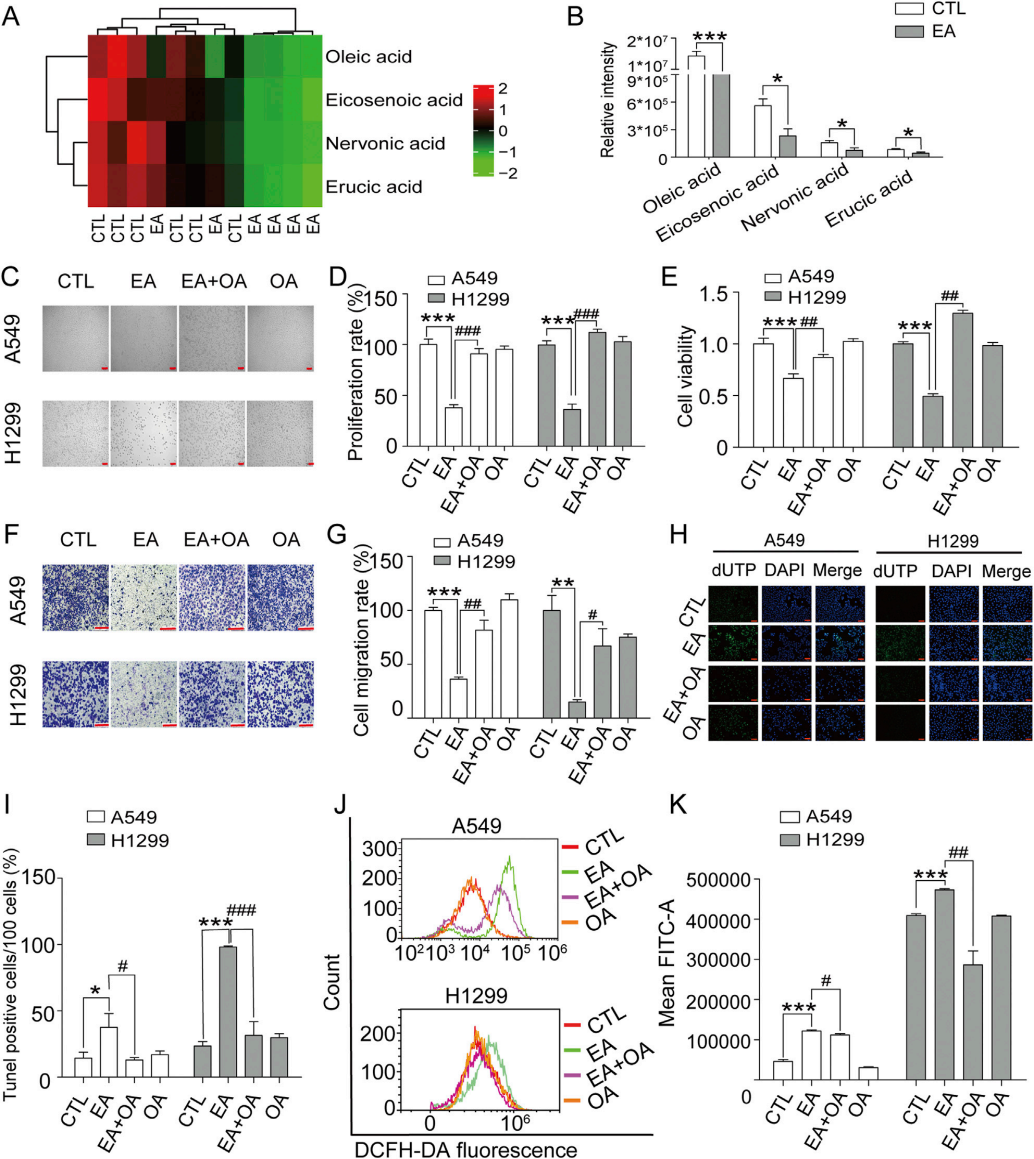
Oleic acid (OA) reverses the inhibitory effects of EA on cell proliferation and migration in human non-small cell lung cancer cells. **(A)** Enrichment of different metabolites in the biosynthesis of unsaturated fatty acids. **(B)** Quantitative analysis of four unsaturated fatty acids by metabolomics. **(C,D)** The effects of EA and OA on cell proliferation of A549 and H1299 cells n = 5. **(E)** The changes of cell viability in A549 and H1299 cells after EA and OA treatment detected by CCK8 n = 6. **(F,G)** The effect of EA or OA on cell migration of A549 and H1299 cells by transwell assay n = 4. **(H,I)** The cell apoptosis after EA or OA treatment detected by Tunel assay in A549 and H1299 cells n = 5. Scale bar = 100 μm **(J,K)** The production of ROS after EA or OA treatment detected by flow cytometry detection in A549 and H1299 cells n = 4. *p < 0.05, **p < 0.01, and ***p < 0.001 compared with the Control group.

Figure 7A shows the complete analysis of the unsaturated fatty acid biosynthetic pathway, presented asa heatmap after EA administration. This examination revealed a significant alteration in the concentrations of four pivotal metabolites: oleic acid, eicosenoic acid, nervonic acid, and erucic acid, all classified as monounsaturated fatty acids. Notably, oleic acid emerged as the most prominently affected, being markedly depleted in NSCLC cells subjected to EA treatment, as shown in Figure 7B. The effect of oleic acid on the therapeutic efficacy of EA against NSCLC was assessed by dividing the cells into four groups: a control group, a group treated with EA, a group treated with EA plus oleic acid, and a group treated with oleic acid alone. Cell imaging results shown in Figures 7C,D, demonstrated that oleic acid supplementation significantly counteracted the EA-mediated reduction in cell count, a result confirmed by CCK8 assay shown in Figure 7E. The effect of oleic acid was also assessed on the migratory effect of these cells through the transwell migration assay. Figures 7F,G show that oleic acid supplementation profoundly reversed the EA-induced suppression of cell migration, suggesting a complex regulatory network involving monounsaturated fatty acids in modulating cancer cell behaviour. Next, the effect of oleic acid on cell apoptosis and ferroptosis induced by EA was investigated by supplementing oleic acid to the cells. Figures 7H,I show a reduction in the proportion of apoptotic cells after oleic acid supplementation compared to the cells treated with EA alone. Moreover, oleic acid supplementation in the effect of EA on cell ferroptosis revealed a significant decrease in ROS production in cells compared to the effect after EA treatment alone (Figures 7J,K). Collectively, these findings revealedthe role of monounsaturated fatty acids, particularly oleic acid, in mitigating the proliferative and migratory effects induced by EA.

### EA regulates the apoptosis and ferroptosis of A549 and H1299 cells by targeting SCD1 expression

**FIGURE 8:**
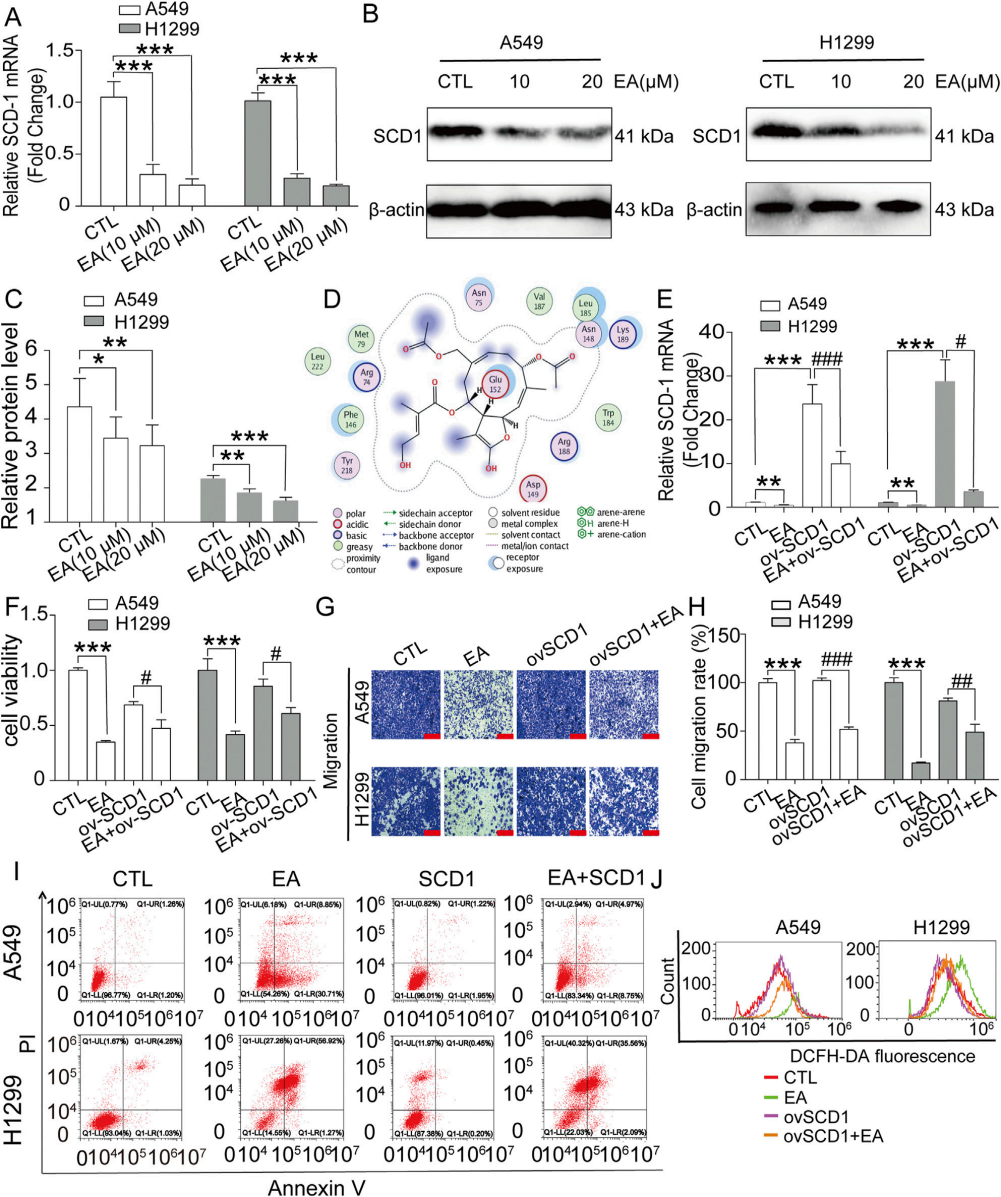
EA induces cell proliferation and migration inhibition of non-small cell lung cancer cells by regulating the expression of SCD1. **(A)** The *SCD-1* mRNA expression detected by qRT-PCR in A549 and H1299 cells n = 6. **(B,C)** Western blot assay of SCD1 protein expression in A549 and H1299 cells treated with different concentrations of EA n = 3. **(D)** Molecular docking of agrimonolide (PubChem CID: 77985550) in the SCD1 protein (protein number: 4zyo) was performing using a computational MOE program. **(E)** The changes of mRNA expression in A549 and H1299 cells after EA administration or overexpression of *SCD1* by qRT-PCR n = 5. **(F)** The viability of A549 and H1299 cells after EA administration or overexpression of *SCD1* by CCK8 assay n = 6. **(G,H)** The effect of EA treatment or *SCD1* overexpression on the migration ability of A549 and H1299 cells by Transwell assay n = 4. Scale bar = 100 μm **(I)** The changes of apoptosis levels in A549 and H1299 cells treated with EA drug or overexpressing *SCD1* by flow cytometry. **(J)** ROS levels in A549 and H1299 cells treated with EA or *SCD1* overexpression detected by flow cytometry. *p < 0.05, **p < 0.01, and ***p < 0.001 compared with the Control group.

Literature research was performed to understand the effect of EA on monounsaturated fatty acid metabolites, revealing that SCD1 is the key factor of unsaturated fatty acid synthesis. SCD1 is a key enzyme in lipid synthesis and catalyses the conversion of saturated fatty acids to monounsaturated fatty acids. The results showed that EA significantly reduced the expression of SCD1 (Figures 8A–C). Molecular docking results showed that EA and SCD1 had strong binding ability (Figure 8D). The regulation of SCD1 in the EA-induced cell growth inhibition and migration inhibition was assessed by constructing a plasmid expressing SCD1, which was transfected into A549 cells and H1299 cells. Figure 8E revealed a marked decrease in the expression of SCD1 after EA treatment and a steep increase after SCD1 gene overexpression, suggesting the successful overexpression of this gene. Next, the effects of SCD1 overexpression on cell proliferation and migration revealed that itn reversed EA-reduced cell viability and migration (Figures 8F–H). This reversing effect was analogous to the effects observed in cell apoptosis and ROS assays, as demonstrated by flow cytometry (Figures 8I,J; Supplementary Figures 1, 3).

### EA exerts its anti-tumour effect on NSCLC by targeting the AMPK signalling pathway

**FIGURE 9:**
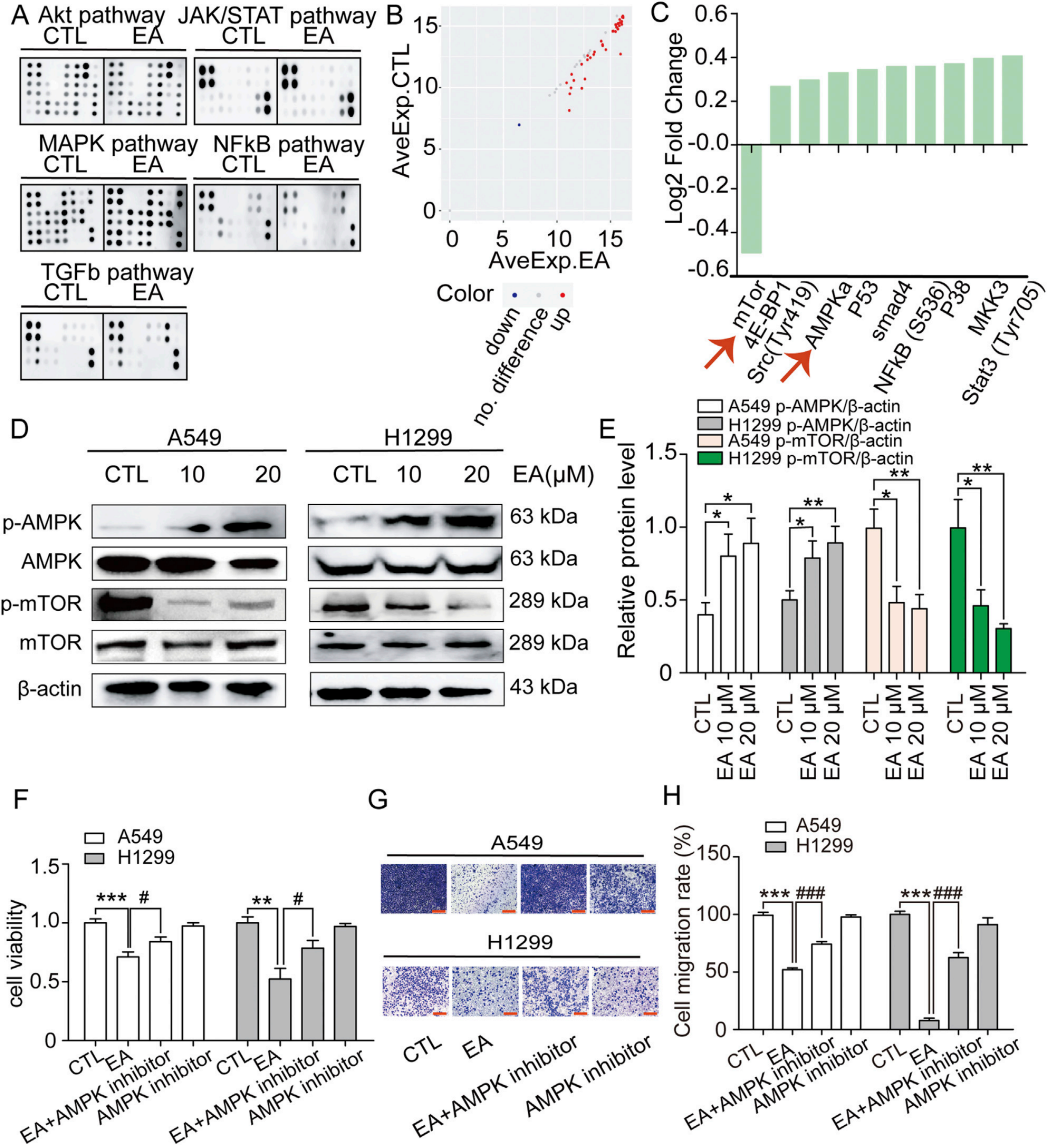
EA activates AMPK-mTOR signal to regulate cell proliferation and migration inhibition of non-small cell lung cancer cells. **(A)** Membranes showing the spots quantified to study the phosphorylation level of 55 proteins in the phosphoprotein array (5 oncogenic pathways; 55 phosphorylated proteins) in response to 24 h treatment EA. **(B)** The scatter plot of differential expression proteins (DEPs). Red presents upregulation, blue presents downregulation, and grey presents no difference. **(C)** Log2 Fold Change of phosphorylation protein level in comparison with the control (DMSO). **(D,E)** Western blot assay of phosphorylation protein expression of AMPK and mTOR in A549 and H1299 cells treated with different concentrations of EA n = 5. **(F)** The viability of A549 and H1299 cells after EA administration or AMPK inhibitor by CCK8 assay n = 6. **(G,H)** The effect of EA treatment or AMPK inhibitor on the migration ability of A549 and H1299 cells by Transwell assay n = 5. Scale bar = 100 μm *p < 0.05, **p < 0.01, and ***p < 0.001 compared with the Control group.

The underlying mechanism used by EA to exert its anti-tumour effect on A549 and H1299 cells was assessed using phosphorylomic analysis, and the results showed 55 phosphorylated proteins in the AKT, JAK/STAT, MAPK, NFκB, and TGF-β pathways, which are all carcinogenic key pathways (Figures 9A,B). However, 41 differentially expressed proteins were found after EA treatment. Among them, the phosphorylation level of one protein such as mTOR, was significantly downregulated, while the phosphorylation level of other 40 proteins was significantly upregulated (Figure 9C). Among these proteins, those that are more related to SCD1 include AMPK and mTOR. Therefore, the expression of AMPK and mTOR was detected and the results showed that the phosphorylation level of AMPK increased significantly while that of mTOR decreased significantly after EA treatment (Figures 9D,E). The result was confirmed using the AMPK inhibitor dorsomorphin dihydrochloride to investigate its effect on cell proliferation and migration. The results showed that dorsomorphin dihydrochloride recoveredthe inhibitory effect of EA in A549 and H1299 cells as shown in Figures 9F–H. Collectively, these results indicated that EA attenuated tumour growth through the AMPK-mTOR-SCD1 signalling pathway.

### EA inhibits NSCLC growth *in vivo*

**FIGURE 10:**
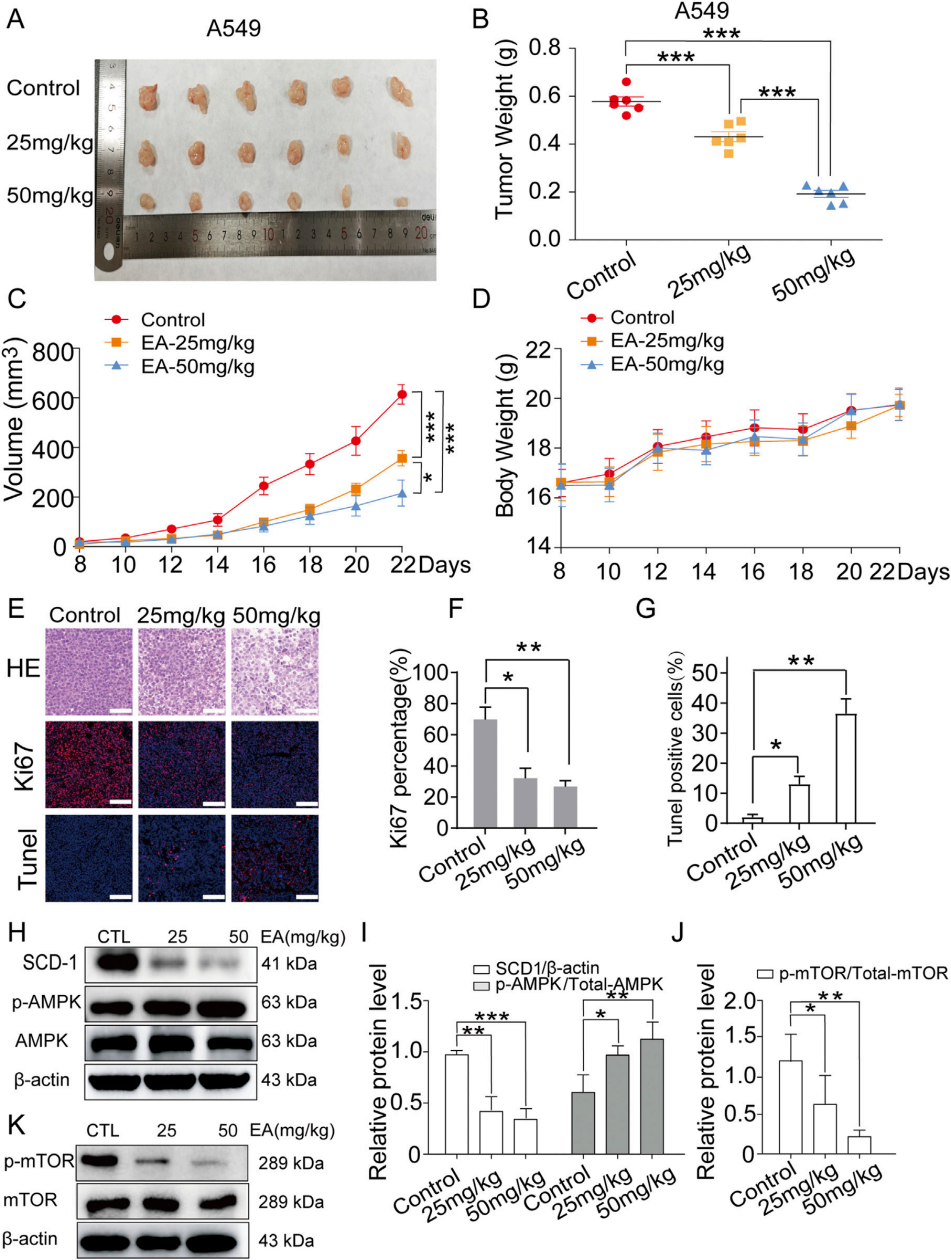
EA suppresses tumor growth in xenograft model of human non-small cell lung cancer cells. **(A)** Photograph of tumors from indicated mice. **(B)** Tumor weight of indicated mice. **(C)** Tumour volume of indicated mice. **(D)** Body weight of indicated mice. *p < 0.05, **p < 0.01, and ***p < 0.001 compared with the Control group. **(E)** H&E, Ki67 and TUNEL staining in indicated tumours. Scale bar = 50 μm. **(F,G)** Quantitative image analysis of Ki67 and Tunel positive cells in **(E)**. **(H–K)** Western blot assay of phosphorylation protein expression of SCD1, AMPK and mTOR in indicated tumours n = 3. *p < 0.05, **p < 0.01, and ***p < 0.001 compared with the Control group.

The anti-tumour effect of EA on NSCLC was investigated using a nude xenograft model of mice, which were treated with EA at different concentrations (25 mg/kg and 50 mg/kg), together with a positive control. Our findings shown in Figure 10A demonstrated that EA exerted an inhibitory effect on tumour growth, highlighting its potential as an anticancer agent. Furthermore, EA significantly attenuated tumour weight and volume in the nude mouse model, corroborating the *in vivo* effect (Figures 10B,C). However, the body weight of nude mice was not significantly affected (Figure 10D). H&E staining further confirmed the aforementioned results (Figure 10E). Furthermore, we performed immunofluorescence (IF) staining for Ki67, TUNEL assay to detect apoptosis, and Western blot to assess AMPK, mTOR, and SCD1 protein expression in tumour tissues derived from model mice. The results showed that Ki67 expression was significantly decreased, while apoptotic cells were significantly increased in the EA group compared with the control group (Figures 10E-G). Western blot analysis revealed that EA treatment upregulated the expression of p-APMK protein while downregulating the expression of p-mTOR and SCD1 proteins (Figures 10H–K). Overall, our extensive research demonstrated the robust inhibitory effects of EA on NSCLC both *in vivo* and *in vitro*, thus providing an encouraging prospect for the development of innovative therapeutic approaches to combat this highly destructive tumour.

## Discussion

**FIGURE 11:**
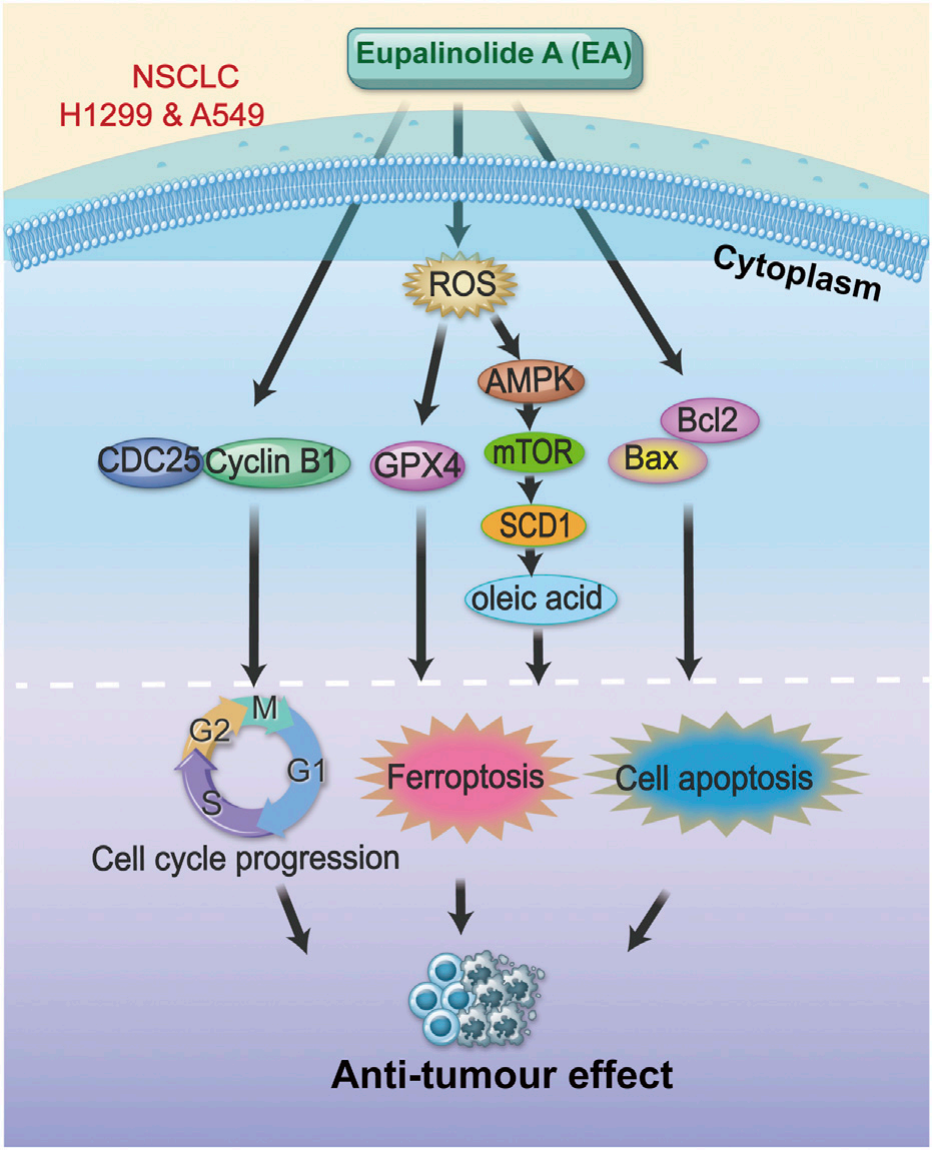
Schematic diagram of the mechanism of EA function.

Despite substantial advances in the treatment of NSCLC in recent years, the prognosis of NSCLC patients is still not ideal (Guo et al., 2024; Wang et al., 2021). Chemotherapy and targeted molecular therapy for lung cancer are usually accompanied by evident side effects, including strong toxicity to liver and kidney, causing vascular damage and drug resistance (Li et al., 2024; Yan et al., 2025). Traditional Chinese herbs are a promising treatment strategy for lung cancer due to their high safety and low cost, which may mitigate the adverse effects of chemotherapeutic drugs (Shi et al., 2022; Wang et al., 2018). Our previous study showed the antitumour effect of EA (Zhang et al., 2022b); however, the role of EA in NSCLC is unclear. This study systematically demonstrated that EA regulated the expression of oleic acid in NSCLC cells by targeting SCD1, inhibited lipid metabolism in NSCLC cells, triggered ferroptosis, promoted cell G2/M cycle arrest, and inhibited cell migration. Therefore, EA showed good antitumor activity and might be an effective adjuvant antitumour drug (Figure 11).

Metabolic dysregulation is an important landmark event in the malignant progression of cancer (Feng et al., 2021). The important link between tumour genetics and metabolic recombination provides a new direction in the treatment of tumours (Tajan and Vousden, 2020). Enhanced lipid synthesis is one of the most significant markers of tumour metabolism, promoting tumour malignant progression (Martin-Perez et al., 2022). Intracellular free fatty acids make cancer cells more aggressive through lipid metabolic remodelling (Choi et al., 2018). Our present results revealed the critical regulatory role of EA in the metabolism of NSCLC cells. Our metabolomics analysis indicated that EA regulated the lipid metabolic pathway, including the biosynthesis of unsaturated fatty acids and fatty acid biosynthesis by reducing oleic acid production, further regulating the malignant behaviour of cells, including apoptosis and migration.

Oleic acid is a monounsaturated fatty acid involved in the development of many solid tumours, including breast cancer, gastric cancer, and prostate cancer (Zhang et al., 2024). Our study demonstrated that increased oleic acid promoted the proliferation and migration of NSCLC cells and inhibited their apoptosis by increasing SCD1 expression. Oleic acid inhibits ferroptosis by regulating the amount of polyunsaturated fatty acids available for oxidation in cell membranes (Magtanong et al., 2019). Our experimental results showed that EA induced ferroptosis by promoting ROS production and inhibiting GPX4 expression. Ferroptosis is a programmed cell death driven by the accumulation of intracellular lipid peroxidation and the excessive accumulation of cellular ROS, which is involved in the occurrence and development of multiple types of cancer including NSCLC (Gao et al., 2024). A series of studies highlighted that the metabolic activity of mitochondria is required for the generation of sufficient ROS to initiate ferroptosis (Gao et al., 2019). When cells start ferroptosis, it leads to alterations in the morphology and cristae structure of mitochondria. TEM revealed small mitochondria with increased mitochondrial membrane density and loss of cristae, while the cell membrane remains intact and the nucleus appears normal in size (Wang H. et al., 2020). Our results demonstrated a significant concentration-dependent increase in ROS levels after EA treatment, the expression of ferroptosis-associated key regulators that counteracted ferroptosis were significantly downregulated, and TEM revealed distinct morphological alterations in EA-treated cells, showing a reduced mitochondrial size associated with an increased membrane density. Tumour cells show a higher dependence on iron than normal cells, meaning that targeting ferroptosis may represent a new therapeutic strategy to combat lung cancer (Wang et al., 2023). A study found that the increased ROS induced by NOX4 is essential for oleic acid promoting the metastasis of colorectal cancer cells (Shen et al., 2020). This work investigated the regulatory role of oleic acid on ferroptosis in NSCLC cells to clarify the underlying mechanism,The results revealed that EA treatment inhibited oleic acid level by reducing SCD1 expression in a concentration-dependent manner, thereby increasing cellular ferroptosis.

SCD1 is an enzyme involved in the synthesis of monounsaturated fatty acids, with a key regulatory role in the PI3K-AKT-mTOR pathway by which it reduces ferroptosis in tumour cells (Yi et al., 2020). AMPK is an important molecule involved in the regulation of lipid metabolism and maintains cellular energy homeostasis (Dong et al., 2024). A previous study showed that AMPK has a dual effect on the regulation of ferroptosis. AMPK-mediated phosphorylation of beclin1 promotes ferroptosis by inhibiting GSH produced in colon cancer cells (Cao et al., 2024). Interestingly, studies found that cancer cells with high AMPK level activation are resistant to ferroptosis; the phosphorylation of ACAC mediated by AMPK inhibits ferroptosis (Zhao et al., 2020; Lee et al., 2020). Our results indicated that EA activated AMPK by the upregulation of ROS, leading to the downregulation of mTOR, further inhibiting the expression of SCD1, thereby promoting ferroptosis.

Nowadays, more and more traditional Chinese medicine monomer compounds have been tested and their role in the treatment of tumours is demonstrated (Li et al., 2021). Studies on EA, as one of the active ingredients of *E. lindleyanum*, are mostly limited on its anti-inflammatory activity, but little is known about its anti-tumour activity. Up to now, there is only one report on the role of EA in cancer (Zhang et al., 2022b). Therefore, our study on the mechanism of EA against NSCLC might provide more theoretical support for its anti-tumour pharmacological action. The most exciting findings of our results was that EA showed a good anti-tumour effect on NSCLC. Our study provides new insights into the regulation of ferroptosis pathway by EA through lipid metabolism, thus inhibiting the malignant behaviour of NSCLC cells, providing a new perspective on its role in NSCLC. However, the target regulators of EA activating the inhibition of lipid metabolism need to be further explored.

In conclusion, our study showed the good antitumor effect of EA by regulating the lipid metabolism pathway through the reduction of oleic acid production in the development and progression of NSCLC. EA regulated the malignant behaviour of NSCLC cells, including the promotion of cell apoptosis and the inhibition of cell proliferation and migration by promoting cellular ferroptosis. Furthermore, this study established a new mechanistic framework for the antitumour effect of EA in NSCLC, identifying the ROS-AMPK-mTOR-SCD1 signalling pathway as a key target, making EA a promising therapeutic candidate for NSCLC.

## Data Availability

The dataset presented in this study can be found in the online repository NCBI, accession number PRJNA1286968 (https://www.ncbi.nlm.nih.gov/bioproject/PRJNA1286968).
