# Supplementary material for: Eupalinolide A inhibits cancer progression and induces ferroptosis and apoptosis by targeting the AMPK/mTOR/SCD1 signalling in non-small cell lung cancer
Source: Front Pharmacol. 2025 Nov 12;16:1649314. doi: 10.3389/fphar.2025.1649314 (PMC12647023; doi:10.3389/fphar.2025.1649314)
Supplement: Supplementary file 1 [file DataSheet1.docx]

**
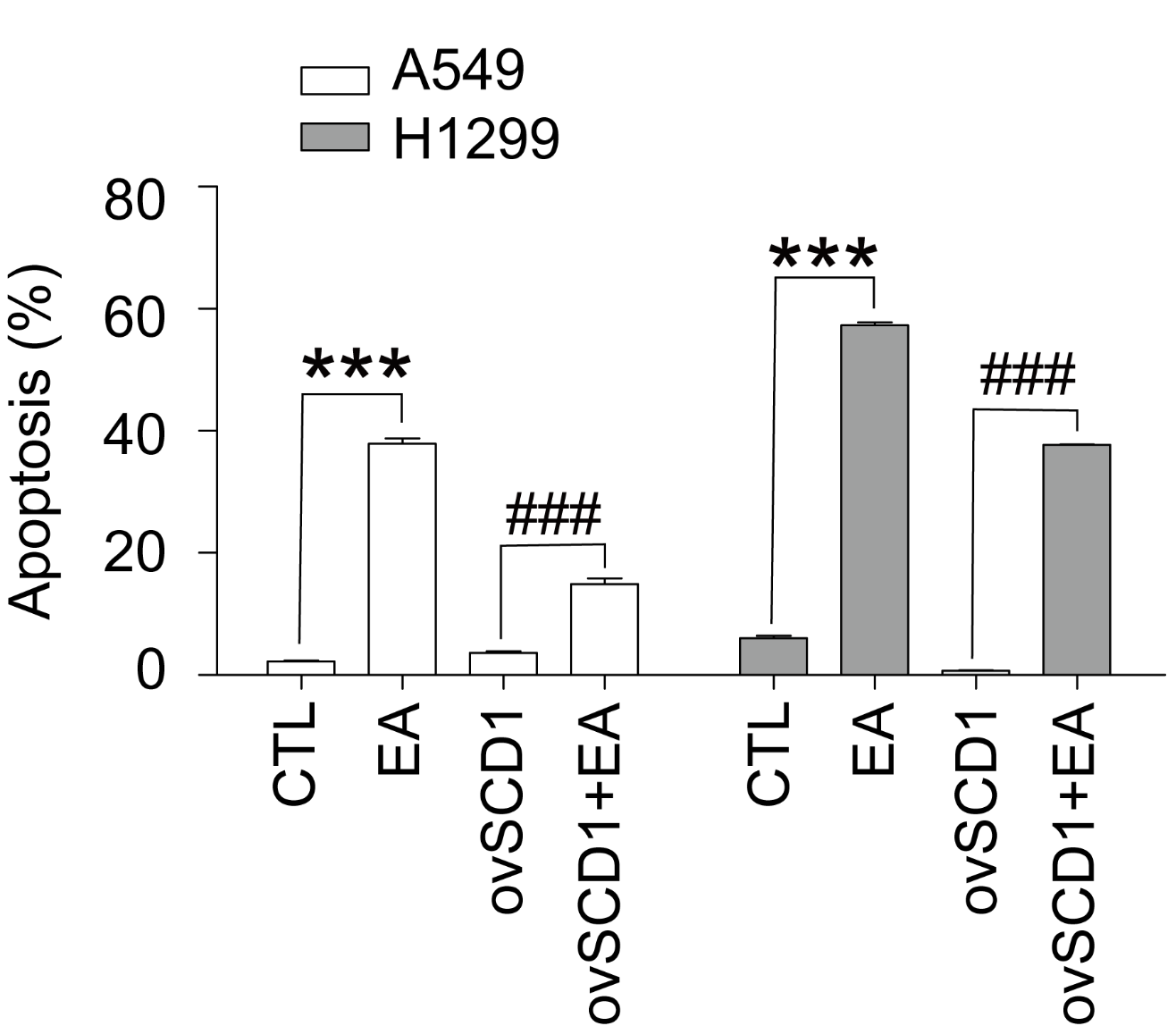
 Figure S1** The data statistics of apoptosis levels in A549 and H1299 cells treated with EA drug or overexpressing *SCD1* by flow cytometry. n=3. **p*<0.05, ***p*<0.01, and ****p*<0.001 compared with the Control group.


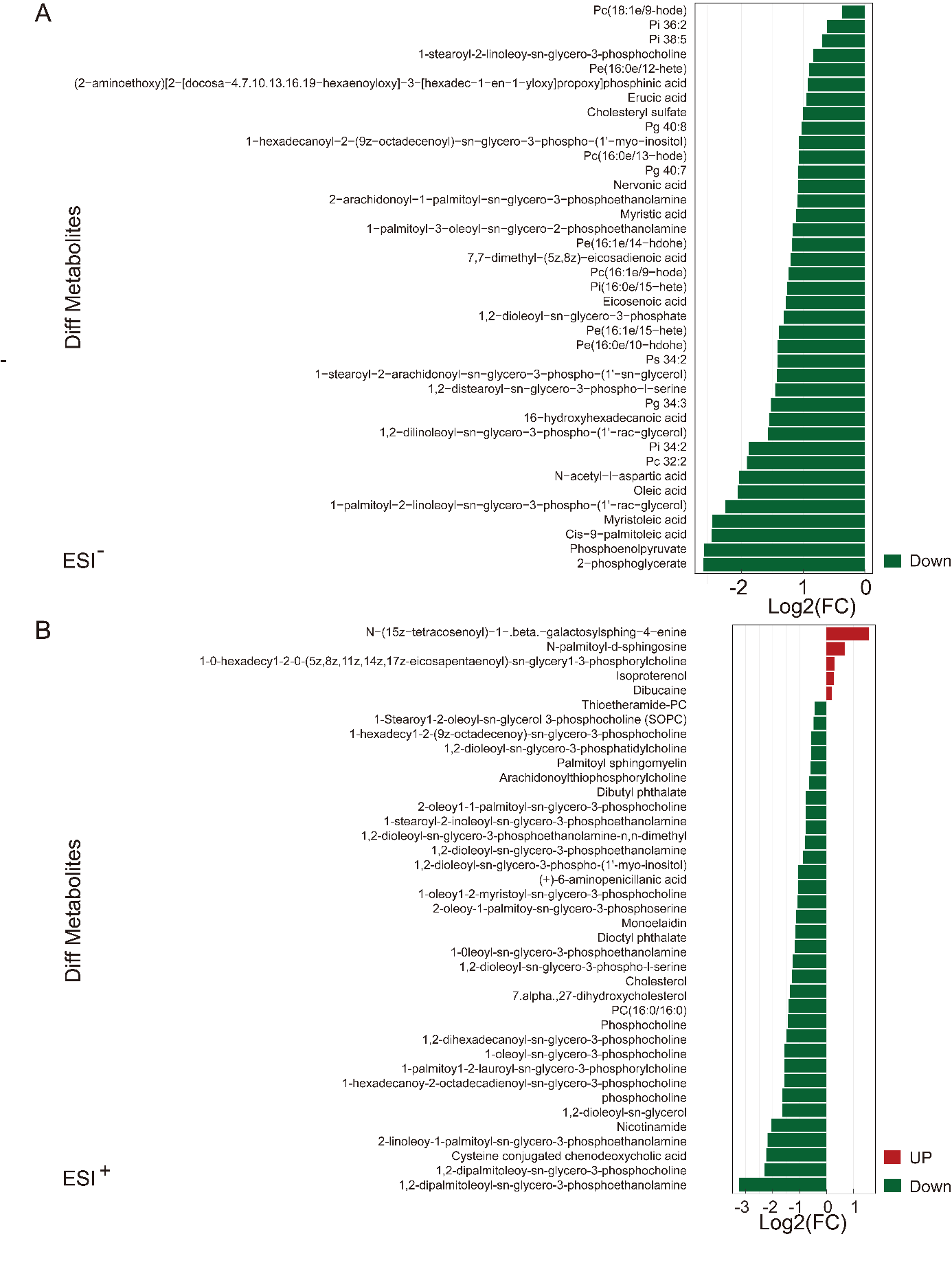
 **Figure S2** The significant expression of different metabolites. (A) Differential expression analysis of significant metabolites in positive ion mode. (B) Differential expression analysis of significant metabolites in negative ion mode.


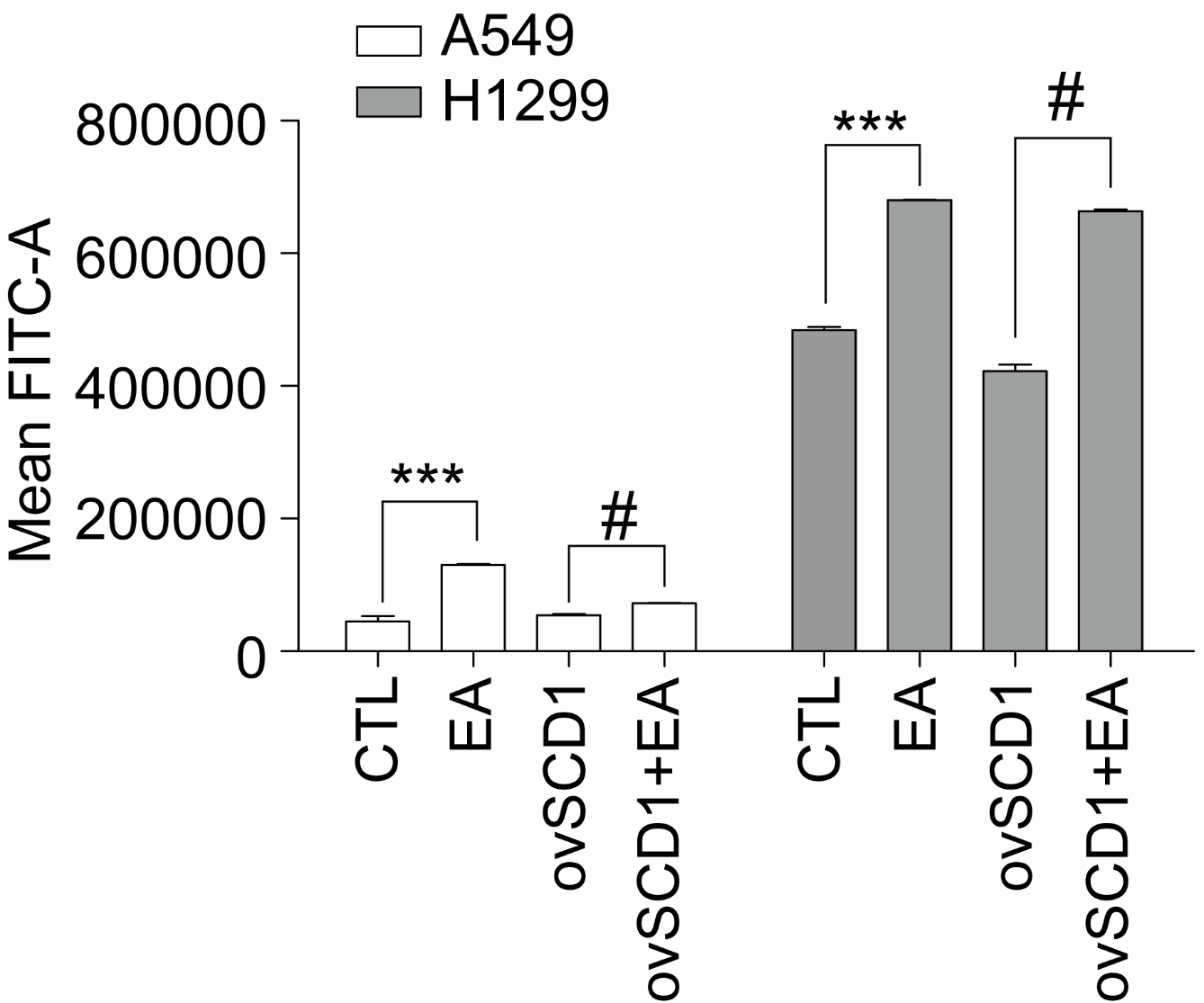


**Figure S3** The data statistics of ROS levels in A549 and H1299 cells treated with EA or *SCD1* overexpression detected by flow cytometry. n=3. **p*<0.05, ***p*<0.01, and ****p*<0.001 compared with the Control group.
